# Mental health patterns of physiotherapists in South Africa during COVID-19

**DOI:** 10.4102/sajp.v79i1.1881

**Published:** 2023-07-06

**Authors:** Nabeelah Bemath, Nicky Israel, Tasneem Hassem

**Affiliations:** 1 School of Anatomical Sciences, Faculty of Health Sciences, University of the Witwatersrand, Johannesburg, South Africa; 2 Department of Psychology, Faculty of Humanities, University of the Witwatersrand, Johannesburg, South Africa

**Keywords:** mental health, COVID-19, frontline, physiotherapists, trend analysis

## Abstract

**Background:**

While attention has been drawn to the impact of the coronavirus disease 2019 (COVID-19) pandemic on the mental health of healthcare workers generally, little is known regarding mental health changes over time in frontline and non-frontline physiotherapists during this period.

**Objectives:**

Our study aimed to investigate differences in mental health trends among frontline and non-frontline physiotherapists across three time periods during the pandemic.

**Method:**

Survey-based data were collected from 366 practising physiotherapists across three time periods during the pandemic (Time 1: *n* = 171; Time 2: *n* = 101; Time 3: *n* = 94). Variations in reported mental health of frontline and non-frontline respondents generally and over time were analysed using comparative statistical techniques and trend analysis.

**Results:**

Frontline physiotherapists reported significantly lower levels of general mental well-being and resilience, and significantly higher levels of burnout and maladaptive strategy use. Only frontline physiotherapists’ general mental well-being and resilience decreased over time, whereas depression decreased over time for both groups. Anxiety decreased over time for non-frontline physiotherapists but initially decreased and then increased for frontline physiotherapists. Burnout increased initially and then decreased for non-frontline physiotherapists.

**Conclusion:**

Varying mental health trends were found between frontline and non-frontline physiotherapists over time. Nuanced mental health interventions that consider the period of the pandemic and degree of exposure are needed.

**Clinical implications:**

Understandings of the mental health trajectories experienced by physiotherapists across the pandemic can inform long-term, targeted interventions that effectively enhance well-being, retention, and sustainability of practitioners, and thus the care delivered, in the healthcare system.

## Introduction

Physiotherapists have played a key role in treating and managing patients during and post the coronavirus disease 2019 (COVID-19) infection (Pegorari et al. 2020; World Physiotherapy 2021). However, the professional practice of physiotherapists and provision of physiotherapy services globally have been challenged and disrupted by the COVID-19 pandemic, raising concerns regarding its impact on physiotherapists’ general well-being and mental health (Minghelli et al. 2020; Pegorari et al. 2020; World Physiotherapy 2021).

Available literature suggests that little is known regarding the psychological well-being of physiotherapists during the pandemic relative to other healthcare workers (Labrague 2021; Moitra et al. 2021; Muller et al. 2020), including within the South African context (Hassem et al. 2022). Studies that are available have found that some physiotherapists experienced emotional stress (Duarte et al. 2022; Tiwari et al. 2021), fear of infection (Palacios-Ceña et al. 2021), increased depression and anxiety symptoms (Samarakoon & Wettasinghe 2022; Yang et al. 2020), and burnout (Pniak et al. 2021) during the pandemic. Socio-demographic factors such as reduced income, a previous diagnosis of depression or anxiety, being female (Duarte et al. 2022), being from an older age bracket, living with a child (Yang et al. 2020), and having more than 10 years of experience (Pniak et al. 2021) were also found to be associated with poorer mental health in physiotherapists during the pandemic.

Studies also suggest that physiotherapists who worked directly with COVID-19 patients may have experienced poorer mental health outcomes (Da Silva Pigati et al. 2022; Hassem et al. 2022; Jácome et al. 2021), in line with findings indicating that the mental health of frontline healthcare workers differed from that of non-frontline healthcare workers during the pandemic (Alshekaili et al. 2020; Carmassi et al. 2020; Moitra et al. 2021; Sheraton et al. 2020). Jácome et al. (2021) found that while physiotherapists who had direct engagement with any patient showed poorer mental health outcomes, working with COVID-19 patients significantly predicted higher levels of personal burnout. A preliminary study in a South African context supported the patterns described here, with the findings indicating that physiotherapists with exposure to COVID-19 patients scored significantly higher on measures of burnout, depression, and anxiety and adopted more maladaptive coping strategies (Hassem et al. 2022).

Despite these findings, minimal research has directly compared the well-being of frontline and non-frontline physiotherapists during the pandemic (Jácome et al. 2021), and little available research appears to have investigated mental health trends among physiotherapists across the pandemic. This is concerning, as repeated cross-sectional (Havaei et al. 2021) and longitudinal studies (Hines et al. 2021; Steinmetz et al. 2021, 2022; Van Steenkiste et al. 2022; Zhou et al. 2021) on the psychological well-being of other healthcare workers have demonstrated that mental health outcomes over the course of the pandemic varied substantially for these groups. Exploring the mental health trends of frontline and non-frontline physiotherapists during the pandemic may thus be an important direction for research, as repeated cross-sectional studies can provide knowledge regarding the changing prevalence of mental health outcomes in a population and identify appropriate interventions needed as a result of this pattern (Sourander et al. 2012).

Gaps in the existing research, the disruptions that physiotherapists experienced because of the COVID-19 pandemic, and the implications this may have for intervention, education, and practice (Minghelli et al. 2020; Pegorari et al. 2020; World Physiotherapy 2021) strongly support further investigating mental health trends in physiotherapists during the pandemic. This is also critical given the unique nature of the South African healthcare context (which is characterised by the increasing quadruple burden of disease specific to the country, systemic and structural inequities, and socioeconomic challenges) and the need for informed and specific interventions tailored to support physiotherapists working in this context (De Villiers 2021; Maphumulo & Bhengu 2019). Our study therefore compared mental health outcomes and explored mental health trends among frontline and non-frontline physiotherapists in South Africa over three time periods during the pandemic, focusing on changes in perceived physical and mental health, depression, anxiety, burnout, resilience, and coping over time.

## Methods

### Research design

A non-experimental, repeated cross-sectional, and survey-based design was employed (Rosenthal & Rosnow 2008). This design repeatedly surveys different groups in a given population and thus allows for changes in prevalence to be estimated at a population level (Sourander et al. 2012). We collected data from three different samples of physiotherapists at three different time periods. Data collection for Time 1 (22 June 2020 – 14 July 2020) occurred during alert level 3 in South Africa. The country was placed on alert level 3 because of a then-moderate spread of infection (South African Government 2020a, 2020b). Data collection for Time 2 (15 October 2020 – 15 November 2020) occurred just prior to the start of the second peak of the pandemic in South Africa, and for Time 3 (28 June 2021 – 20 July 2021), data collection occurred during the third peak of the pandemic in South Africa (Centre for Respiratory Diseases and Meningitis NICD-NHLS 2021). During each period, online surveys consisting of a range of mental health measures were distributed to as many qualified physiotherapists in South Africa as possible with an invitation to participate in our study on a voluntary basis. To ensure that participants were independent across time periods, they were requested in the survey to provide the first five digits of their cell phone number. This was used to identify and remove the second and/or third response from the same participant across the different time periods.

### Sample and sampling

In alignment with our study design, a total sample of 366 independent responses from qualified physiotherapists in South Africa was obtained across the three time periods (i.e., different samples of physiotherapists were obtained for each time period) using a non-probability convenience volunteer sampling approach (Rosenthal & Rosnow 2008). In each time period, the sample was categorised based on whether they were exposed to COVID-19 patients in their professional practice or not (i.e., frontline and non-frontline groups). The sample sizes for the groups were 171 (69 frontline, 102 non-frontline) in Time 1, 101 (58 frontline, 43 non-frontline) in Time 2, and 94 (56 frontline, 38 non-frontline) in Time 3. The demographic characteristics of the sample per time and frontline group are reported in Table 1. Most participants in each group identified as being female, Christian, married, held a bachelors’ degree as their highest level of education, spoke English as a home language, and lived with a partner or partner and children.

**TABLE 1:** Sample demographics per time and COVID-19 patient exposure group (*n* = 366).

Variables	Time 1		Time 2		Time 3	
	FL	NFL	FL	NFL	FL	NFL
**Group size**						
*n*	69	102	58	43	56	38
**Age**						
Mean	33.75	39.56	38.21	43.83	36.79	45.24
s.d.	9.97	11.55	10.14	11.71	11.02	13.70
Range	22–62	22–70	24–62	25–70	22–65	20–74
Missing						
*n*	2	-	-	1	-	-
%	2.9	-	-	2.3	-	-
**Years practice**						
Mean	11.17	16.63	15.24	20.63	14.05	22.86
s.d.	10.13	11.49	9.89	12.12	11.14	13.26
Range	0.5–42	0.5–49	2–40	1–50	1–44	0.5–52
Missing						
*n*	-	-	-	-	-	1
%	-	-	-	-	-	2.6
**Gender**						
Female						
*n*	67	96	56	37	51	36
%	97.1	94.1	96.6	86.0	91.1	94.7
Male						
*n*	1	6	2	5	5	2
%	1.4	5.9	3.4	11.6	8.9	5.3
Missing						
*n*	1	-	-	1	-	-
%	1.4	-	-	2.3	-	-
**Education**						
Bachelors						
*n*	58	82	45	35	45	27
%	84.1	80.4	77.6	81.4	80.4	71.1
Masters						
*n*	11	18	12	6	10	9
%	15.9	17.6	20.7	14.0	17.9	23.7
PhD						
*n*	-	-	-	1	1	-
%	-	-	-	2.3	1.8	-
Missing						
*n*	-	2	1	1	-	2
%	-	2.0	1.7	2.3	-	5.3
**Home language**						
Afrikaans						
*n*	28	30	24	8	20	10
%	40.6	29.4	41.4	18.6	35.7	26.3
English						
*n*	35	68	26	33	32	28
%	50.7	66.7	44.8	76.7	57.1	73.7
German						
*n*	2	-	1	1	-	-
%	2.9	-	1.7	2.3	-	-
isiXhosa						
*n*	2	1	2	-	1	-
%	2.9	1.0	3.4	-	1.8	-
isiZulu						
*n*	-	-	1	-	-	-
%	-	-	1.7	-	-	-
Sepedi						
*n*	1	3	1	-	-	-
%	1.4	2.9	1.7	-	-	-
Sesotho						
*n*	-	-	1	-	-	-
%	-	-	1.7	-	-	-
Other						
*n*	-	-	1	1	2	-
%	-	-	1.7	2.3	3.6	-
Missing						
*n*	1	-	1	-	1	-
%	1.4	-	1.7	-	1.8	-
**Religion**						
No religion						
*n*	7	12	3	3	6	5
%	10.1	11.8	5.2	7.0	10.7	13.2
Christianity						
*n*	51	83	48	36	43	28
%	73.9	81.4	82.8	83.7	76.8	73.7
Hinduism						
*n*	3	3	3	1	2	2
%	4.3	2.9	5.2	2.3	3.6	5.3
Islam						
*n*	6	2	1	1	5	-
%	8.7	2.0	1.7	2.3	8.9	-
Judaism						
*n*	1	1	1	1	-	3
%	1.4	1.0	1.7	2.3	-	7.9
Unspecified						
*n*	1	1	2	1	-	-
%	1.4	1.0	3.4	2.3	-	-
Missing						
*n*	-	-	-	-	-	-
%	-	-	-	-	-	-
**Relationship status**						
Married						
*n*	30	65	38	31	36	29
%	43.5	63.7	65.5	72.1	64.3	76.3
Relationship						
*n*	23	17	12	5	12	6
%	33.3	16.7	20.7	11.6	21.4	15.8
Neither						
*n*	16	20	8	7	8	3
%	23.2	19.6	13.8	16.3	14.3	7.9
Missing						
*n*	-	-	-	-	-	-
%	-	-	-	-	-	-
**Number of children**						
0 (None)						
*n*	42	44	24	15	25	8
%	60.9	43.1	41.4	34.9	44.6	21.1
1						
*n*	7	18	8	4	9	3
%	10.1	17.6	13.8	9.3	16.1	7.9
2						
*n*	14	31	14	18	14	17
%	20.3	30.4	24.1	41.9	25.0	44.7
3						
*n*	3	7	9	5	7	7
%	4.3	6.9	15.5	11.6	12.5	18.4
4 or more						
*n*	2	1	3	1	1	3
%	2.9	1.0	5.2	2.3	1.8	7.9
Missing						
*n*	1	1	-	-	-	-
%	1.4	1.0	-	-	-	-
**Living situation**						
Alone						
*n*	10	10	9	8	7	5
%	14.5	9.8	15.5	18.6	12.5	13.2
Partner						
*n*	23	32	11	13	18	8
%	33.3	31.4	19.0	30.2	32.1	21.1
Partner and children						
*n*	20	42	30	17	24	23
%	29.0	41.2	51.7	39.5	42.9	60.5
Children						
*n*	1	3	2	1	1	1
%	1.4	2.9	3.4	2.3	1.8	2.6
Close family						
*n*	11	14	4	3	5	1
%	15.9	13.7	6.9	7.0	8.9	2.6
Relatives						
*n*	4	1	2	1	1	-
%	5.8	1.0	3.4	2.3	1.8	-
Missing						
*n*	-	-	-	-	-	-
%	-	-	-	-	-	-
**Medication**						
Yes						
*n*	24	35	25	23	27	17
%	34.8	34.3	43.1	53.5	48.2	44.7
No						
*n*	45	66	33	20	29	21
%	65.2	64.7	56.9	46.5	51.8	55.3
Missing						
*n*	-	1	-	-	-	-
%	-	1.0	-	-	-	-
**Chronic condition**						
Yes						
*n*	14	23	21	18	25	12
%	20.3	22.5	36.2	41.9	44.6	31.6
No						
*n*	55	79	37	25	31	26
%	79.7	77.5	63.8	58.1	55.4	68.4
Missing						
*n*	-	-	-	-	-	-
%	-	-	-	-	-	-

FL, frontline; NFL, non-frontline; s.d., standard deviation.

### Instruments

The online survey comprised the following instruments: a demographic questionnaire, global health indicators, various mental health scales, and six open-ended questions.

#### Demographic questionnaire

This questionnaire captured participants’ sociodemographic background, specifically age, gender, home language, highest level of qualification, years of professional practice, religion, relationship status, number of children, living arrangements, chronic medical conditions, whether participants were on medication at the time of the survey, and exposure to patients with COVID-19.

#### Global health indicators

One item from the Global Physical Health Scale (GPH-4) and one item from the Global Mental Health Scale (GMH-4) (Hays et al. 2017) were used to establish self-reported physical and mental health levels among participants. The item-response format followed a five-point scale ranging from ‘poor’ to ‘excellent’. As single items were used, reliability could not be established for our study; however, several versions of the GPH and the GMH scales have been shown to be reliable (Hays et al. 2017).

#### Hospital Anxiety and Depression Scale

The Hospital Anxiety and Depression Scale (HADS) established levels of anxiety and depression among participants. The measure has two subscales comprising seven items each (Snaith 2003). Each item has a four-point response format with unique anchors (Snaith 2003). The validity of the HADS has been demonstrated across various settings (Bjelland et al. 2002; Snaith 2003). Cronbach’s alpha for the anxiety and depression subscales were 0.72 and 0.77, respectively, indicating moderate internal consistency reliability (Murphy & Davidshofer 2004).

#### Burnout Measure – Short Version

The Burnout Measure – Short Version (BMS) was used to ascertain participants’ burnout with reference to emotional, mental, and physical exhaustion (Malach-Pines 2005). The BMS has 10 items with a seven-point response format (‘never’ to ‘always’) and has been shown to be psychometrically sound (Fatoki 2019; Malach-Pines 2005). The Cronbach’s alpha was 0.93 indicating extremely high internal consistency reliability (Murphy & Davidshofer 2004).

#### The Connor-Davidson Resilience Scale-10

The Connor-Davidson Resilience Scale-10 (CD-RISC-10) measured participants’ resilience (Connor & Davidson 2003; Vaishnavi, Connor & Davidson 2007). It is a 10-item scale with a four-point response format ranging from ‘not true at all’ to ‘true nearly all of the time’. The CD-RISC-10 has been validated (Vaishnavi et al. 2007). The Cronbach’s alpha for the CD-RISC-10 was 0.90, indicating extremely high internal consistency reliability (Murphy & Davidshofer 2004).

#### The Brief Coping Orientation to Problems Experienced (COPE) Inventory

This scale was used to assess coping approaches among participants (Carver 1997). It consists of 28 items, each with a four-point response format (‘I haven’t been doing this’ to ‘I’ve been doing this a lot’). The items are divided into 14 subscales. The Brief COPE Inventory has been found to be reliable and closely replicates the factor structure of the original inventory (Carver 1997). We collapsed the 14 subscales into two larger coping indices, namely adaptive and maladaptive coping strategies, and Cronbach’s alpha for these indices were 0.85, indicating high internal consistency reliability, and 0.76, indicating moderate internal consistency reliability, respectively (Murphy & Davidshofer 2004).

### Data analysis

Internal consistency reliability estimates and descriptive statistics were calculated for all the demographic and main variables. A series of independent *t*-tests were then carried out to establish whether there were significant differences in the mental health profiles of those physiotherapists in the full sample who had exposure to COVID-19 positive patients (the frontline group) and those who had not (the non-frontline group). This was followed by a series of one-way analysis of variance (ANOVA) to establish whether there were significant differences in the mental health profiles of the physiotherapists who responded across the three data collection periods; Tukey HSD post hoc tests were calculated where significant differences were identified. In any instances where the assumption of homogeneity of variance was violated, Welch’s ANOVA and Games-Howell post hoc analyses were utilised instead. Linear and quadratic contrasts were then calculated across the time periods to establish whether there were any significant patterns in the scores obtained over time (trend analysis). The completion rate for those who accessed the survey was 85.8%, and missing values were managed using a combination of imputation and deletion.

### Ethical considerations

Ethical clearance for our study was granted by the Human Research Ethics Committee (Medical) of the University of the Witwatersrand (M200461). Permission to conduct research at local government hospitals was obtained. The survey was distributed using an online survey platform. The participation information sheet and questionnaire link were distributed through social media and shared with employees at local hospitals and members of the South African Society of Physiotherapy. The participation information sheet specified that completion of the questionnaire would be taken as participants’ consent to participate. An anonymised data set was used to run the analyses and any identifying information was stored separately in password protected storage spaces accessible only to the research team.

## Results

In the first stage of the analysis, comparisons were carried out to establish whether there were significant differences in the mental health profiles of the physiotherapists in the frontline group and those in the non-frontline group regardless of the time period. The results indicated that there were significant differences between these groups for levels of general mental well-being (*t*_363_ = –3.93; *p* = 0.000; *d* = –0.41) and resilience (*t*_357_ = –2.20; *p* = 0.029; *d* = –0.23) – these were significantly lower for the frontline group although the effect sizes, representing practical significance, were small. Significant differences were also identified between the groups for levels of burnout (*t*_355_ = 4.88; *p* = 0.000; *d* = 0.52) and maladaptive coping strategy use (*t*_351_ = 4.36; *p* = 0.000; *d* = 0.35) – these were significantly higher for the frontline group, although the effect sizes were moderate and small, respectively. No significant differences were identified between the groups for levels of general physical health, depression, anxiety, or adaptive coping strategy use.

Comparisons between the three time periods in the frontline group indicated that there were significant differences among the time periods for levels of general mental well-being (*F*_(2;180)_ = 3.28; *p* = 0.040; *η*^2^ = 0.035) – this was significantly lower for Time 3 compared with Time 1 (see Table 2). These data also followed a significant linear trend (*t*_180_ = –2.37; *p* = 0.019), suggesting that there was a steady reduction in self-reported general mental well-being for the frontline group over time. In contrast, there were no significant differences in levels of self-reported general mental well-being across the three time periods for the non-frontline group and no significant trends in the data (see Table 3).

**TABLE 2:** Frontline group: Comparisons across time periods (*n* = 183).

Variables	Time	*n*	Mss.	M	s.d.	*df*1	*df*2	*F*	η^2^	Pairs	M. diff.
Physical well-being	1	69	-	3.84	0.797	2	180	0.389	0.004	1-2	-
	2	58	-	3.78	0.750	-	-	-	-	1-3	-
	3	56	-	3.71	0.847	-	-	-	-	2-3	-
Mental well-being	1	69	-	3.26	1.010	2	180	3.281*	0.035	1-2	0.037
	2	58	-	3.22	1.009	-	-	-	-	1-3	0.439*
	3	56	-	2.82	1.081	-	-	-	-	2-3	0.403
Resilience	1	69	-	27.70	6.629	2	175	5.286**	0.057	1-2	−0.541
	2	55	3	28.24	5.796	-	-	-	-	1-3	3.048*
	3	54	2	24.65	6.256	-	-	-	-	2-3	3.588**
Depression†	1	69	-	12.36	1.978	2	98	78.603***	0.400	1-2	6.121***
	2	58	-	6.24	4.366	-	-	-	-	1-3	5.023***
	3	56	-	7.34	3.589	-	-	-	-	2-3	−1.098
Anxiety†	1	69	-	11.23	2.450	2	103	5.688**	0.060	1-2	2.215**
	2	58	-	9.02	4.718	-	-	-	-	1-3	0.982
	3	56	-	10.25	3.714	-	-	-	-	2-3	−1.233
Burnout	1	68	1	35.51	10.914	2	173	1.102	0.013	1-2	-
	2	55	3	35.27	13.289	-	-	-	-	1-3	-
	3	53	3	38.30	11.590	-	-	-	-	2-3	-
Adaptive coping	1	69	-	30.87	8.165	2	180	1.382	0.015	1-2	-
	2	58	-	32.72	10.516	-	-	-	-	1-3	-
	3	56	-	29.91	9.014	-	-	-	-	2-3	-
Maladaptive coping	1	69	-	4.78	4.029	2	180	1.245	0.014	1-2	-
	2	58	-	4.76	4.390	-	-	-	-	1-3	-
	3	56	-	5.82	4.037	-	-	-	-	2-3	-

Note: Bold data is significant.

Mss., missing; M, mean; s.d., standard deviation; *df*, degree of freedom; *F*, test statistic; M. diff., mean difference.

* , *p* < 0.05;

** , *p* < 0.01;

*** , *p* < 0.001.

† , Welch’s ANOVA.

**TABLE 3:** Non-frontline group: Comparisons across time periods (*n* = 183).

Variables	Time	*n*	Mss.	M	s.d.	*df*1	*df*2	*F*	η^2^	Pairs	M. diff.
Physical well-being	1	102	-	3.93	0.812	2	179	0.023	0.000	1-2	-
	2	43	-	3.91	0.895	-	-	-	-	1-3	-
	3	37	1	3.95	0.848	-	-	-	-	2-3	-
Mental well-being	1	102	-	3.66	0.970	2	179	1.780	0.020	1-2	-
	2	43	-	3.30	1.124	-	-	-	-	1-3	-
	3	37	1	3.51	1.121	-	-	-	-	2-3	-
Resilience†	1	102	-	28.84	5.743	2	70	0.592	0.008	1-2	-
	2	42	1	28.38	7.322	-	-	-	-	1-3	-
	3	37	1	27.38	7.485	-	-	-	-	2-3	-
Depression†	1	102	-	12.40	1.667	2	60	157.824**	0.654	1-2	**7.030** **
	2	43	-	5.37	4.135	-	-	-	-	1-3	**8.024** **
	3	37	1	4.38	3.085	-	-	-	-	2-3	0.994
Anxiety†	1	102	-	10.24	2.347	2	62	4.652*	0.049	1-2	1.421
	2	43	-	8.81	5.430	-	-	-	-	1-3	**1.857** *
	3	37	1	8.38	3.897	-	-	-	-	2-3	0.436
Burnout	1	102	-	29.87	11.629	2	178	2.278	0.025	1-2	-
	2	42	1	32.98	14.381	-	-	-	-	1-3	-
	3	37	1	27.16	10.658	-	-	-	-	2-3	-
Adaptive coping	1	102	-	29.84	9.709	2	179	1.562	0.017	1-2	-
	2	43	-	32.84	11.506	-	-	-	-	1-3	-
	3	37	1	32.08	9.912	-	-	-	-	2-3	-
Maladaptive coping	1	102	-	3.50	3.338	2	179	0.834	0.009	1-2	-
	2	43	-	4.30	3.713	-	-	-	-	1-3	-
	3	37	1	3.81	3.340	-	-	-	-	2-3	-

Note: Bold data is significant.

Mss., missing; M, mean; s.d., standard deviation; *df*, degree of freedom; *F*, test statistic; M. diff., mean difference.

* , *p* < 0.05;

** , *p* < 0.001.

† , Welch’s ANOVA.

Similarly, there were no significant differences in levels of resilience between the three time periods for the non-frontline group and no significant trends in the data; however there were significant differences for the frontline group (*F*_(2;175)_ = 5.29; *p* = 0.006; *η*^2^ = 0.057). Post hoc analysis suggested that resilience was significantly lower for Time 3 compared with Time 1 and Time 2 (see Table 2). These data also followed both a significant linear trend (*t*_175_ = –2.68; *p* = 0.008) and a significant quadratic trend (*t*_175_ = –2.03; *p* = 0.044) – these suggested that in the sample, resilience trended significantly downwards over time for the frontline group.

There were significant differences in levels of depression between the time periods for both the frontline group (*F*_(2;98)_ = 78.60; *p* = 0.000; *η*^2^ = 0.400) and the non-frontline group (*F*_(2;60)_ = 157.82; *p* = 0.000; *η*^2^ = 0.654). For both groups, depression was significantly higher at Time 1 compared with Time 2 and Time 3 (see Table 2 and Table 3). In both groups, the data followed both a significant linear trend (frontline: *t*_81_ = –9.38; *p* = 0.000; non-frontline: *t*_44_ = –15.04; *p* = 0.000) and a significant quadratic trend (frontline: *t*_82_ = 5.70; *p* = 0.000; non-frontline: *t*_57_ = 4.41; *p* = 0.000) – these suggested that in the sample, depression trended significantly downwards over time for both the frontline group and the non-frontline group.

Significant differences in levels of anxiety between the time periods were also identified for both the frontline group (*F*_(2;103)_ = 5.69; *p* = 0.005; *η*^2^ = 0.060) and the non-frontline group (*F*_(2;62)_ = 4.65; *p* = 0.013; *η*^2^ = 0.049). In the frontline group, anxiety was significantly higher at Time 1 compared with Time 2 (see Table 2). These data also followed a significant quadratic trend (*t*_82_ = 2.52; *p* = 0.014), suggesting that anxiety initially decreased but then increased again over time for the frontline group. In contrast, anxiety was significantly lower for Time 3 compared with Time 1 for the non-frontline group (see Table 3) and the data followed a significant linear trend (*t*_46_ = –2.73; *p* = 0.009), suggesting that there was a steady reduction in levels of anxiety for the non-frontline group over time.

Although there were no significant differences in burnout levels between the time periods for either group, the data for burnout for the non-frontline group did follow a significant quadratic trend in the sample (*t*_178_ = –2.02; *p* = 0.045). This suggests that burnout initially increased but then decreased over time for the non-frontline group.

## Discussion

Our study was a novel investigation of mental health trends among frontline and non-frontline physiotherapists in South Africa during three time points in the COVID-19 pandemic. Work of this nature is important as tracking the mental health of physiotherapists through the progression of the pandemic may yield valuable insights for effective future interventions to improve well-being, support long-term occupational sustainability, and reduce turnover (De Kock et al. 2021; Marvaldi et al. 2021).

The initial comparison of frontline and non-frontline physiotherapists, irrespective of time, identified significantly lower levels of general mental health and resilience in the frontline group, reflecting similar findings to other studies of frontline healthcare workers during the pandemic (Alshekaili et al. 2020; Cai et al. 2020; De Kock et al. 2021). Frontline physiotherapists in the sample also reported significantly higher levels of burnout and maladaptive coping strategy use, in line with international findings (Jácome et al. 2021; Pniak et al. 2021). This may be because of the increase in workplace demands and workload experienced by frontline healthcare workers during the pandemic (De Kock et al. 2021; Søvold et al. 2021; Steinmetz et al. 2021). The significantly greater use of maladaptive coping strategies adds to previous findings that frontline physiotherapists chose not to speak about their experiences during the pandemic to cope with these (Palacios-Ceña et al. 2021). This finding is particularly noteworthy as there appears to be very limited research available regarding negative coping strategies implemented by physiotherapists to manage the effects of the pandemic; despite the potential value of this information as a basis for intervention (Joshi et al. 2021).

No significant differences between the groups were found on measures of general physical health, depression, anxiety, and adaptive coping strategy use. This contrasts with findings in both physiotherapist (Jácome et al. 2021) and other healthcare worker samples (Alshekaili et al. 2020; Moitra et al. 2021) except for depression (Jácome et al. 2021). Contextual differences may account for these discrepancies as it is possible that the impact of COVID-19 on healthcare workers may vary across contexts and be based on situational characteristics (Cabarkapa et al. 2020). Differences in sample characteristics across the various studies may also account for these contrasting findings.

A closer examination of the trends within the frontline and non-frontline groups in our study revealed more nuanced patterns emerging between the groups over time. Based on the trend analysis, frontline physiotherapists experienced a significant reduction in general mental health and resilience over time – this was not observed in the non-frontline group. This concurs with previous longitudinal (Hines et al. 2021; Steinmetz et al. 2022; Zhou et al. 2021) and cross-sectional studies (Jácome et al. 2021; Yang et al. 2020). These findings may reflect differences in the degree of repeated exposure to traumatic events occasioned by frontline healthcare work, particularly during an ongoing emergency situation (Hooper et al. 2021; Kira et al. 2021; Søvold et al. 2021); heightened fear and awareness of contracting and spreading the virus because of direct contact with COVID-19 patients; the implementation of complex safety procedures, and ongoing disruption to regular work routines and the work environment (De Kock et al. 2021; Palacios-Ceña et al. 2021).

For the frontline group, both general mental health and resilience at Time 3 were significantly lower when compared with Time 1 and Time 2. This may be accounted for by the spread of the more infectious and severe Delta-B.1.617.2 coronavirus variant that occurred at Time 3 and the subsequent possible uncertainty and concerns experienced by frontline workers (Abdool Karim & Baxter 2022). This finding may also lend support to the proposition that COVID-19 constitutes a source of continuous and cumulative traumatic stress, thereby reducing coping capacity and depleting personal resources over time (Kira et al. 2021; Marvaldi et al. 2021). These findings suggest that there is a need for workplaces to create spaces and opportunities that foster frontline physiotherapists’ general mental health and resilience, including their management of chronic and/or cumulative traumatic stress (De Kock et al. 2021; Kira et al. 2021). Similarly, physiotherapy training programmes should incorporate self-care management strategies to equip future frontline workers.

Depression levels in both the exposure and non-exposure groups trended significantly downwards over time, directly contradicting findings from other studies (Joshi et al. 2021; Steinmetz et al. 2022). It is possible that depressive symptoms may have been underreported in the sample because of perceived stigma as well as an emphasis on reporting the somatic symptoms of depression (Alshekaili et al. 2020; Crawford & Lipsedge 2006; Mosotho et al. 2008; Sorsdahl et al. 2010). However, the findings may also point to a unique trend in South African physiotherapists – this merits further exploration and highlights the potential value of context-specific research as a basis for intervention and training.

Levels of anxiety in frontline physiotherapists followed a significant quadratic trend, where anxiety decreased initially but then increased. For non-frontline physiotherapists, the trend was linear where anxiety significantly decreased over time. The initially higher levels of anxiety at Time 1 for both groups may have been because of general uncertainty regarding COVID-19 and its effects, as well as fear of infection for oneself and close others (De Kock et al. 2021; Pedersini et al. 2021). Increased availability of information about the virus and the development of effective treatment protocols may have lessened this over time. The increase in anxiety at Time 3 for the frontline group may be explained by contextual factors such as the peak of the Delta variant in South Africa during this time and its impact (Centre for Respiratory Diseases and Meningitis NICD-NHLS 2021). This is similar to the results from a longitudinal study conducted in Argentina (Steinmetz et al. 2022). This finding suggests that interventions addressing anxiety may be particularly needed during peak periods of the pandemic for frontline physiotherapists. Investigating the efficacy of longer-term strategies to facilitate the recognition of periods of high stress and resultant changes in self-care strategies and organisational support to accommodate these may also be valuable (De Kock et al. 2021; Marvaldi et al. 2021).

No significant differences in burnout levels were found across the different times for either group. There was, however, a significant quadratic trend in burnout levels for the non-frontline group; these initially increased between Time 1 and Time 2, and then decreased between Time 2 and Time 3. This trend may be explained by changes in working conditions faced by physiotherapists during the pandemic such as loss of income because of reduced patient numbers, forced changes to treatment modality and processes and the implementation of complicated new safety protocols (Jácome et al. 2021; Pniak et al. 2021; World Physiotherapy 2021). These disruptions may have been especially salient near the start of the pandemic for the non-frontline group, most likely to be working in private practice, leading to initially higher levels of reported burnout. The psychological stress created by these challenges may have dissipated over time as physiotherapists adjusted to their new working circumstances and as lockdown levels eased during the later timeframes (South African Government 2020a,b), possibly accounting for the observed reduction across Times 2 and 3. This finding highlights the importance of tracking changes in mental health trajectories for physiotherapists working in different circumstances and times as a basis for designing both situated and context-specific organisational support programmes as well as self-care training, including identifying when particular types of intervention might be most relevant for each group across the course of a pandemic (De Kock et al. 2021; Søvold et al. 2021).

Our study has several limitations. As we used a repeated cross-sectional design, the trends observed may reflect individual differences in sample characteristics among the various groups. Furthermore, our study may be vulnerable to confounding variables because of its cross-sectional nature. Variables such as participants’ infection status, vaccination status and nature of funding (private or state) were not measured and may be potential confounders. Definitive conclusions regarding differences in mental health indicators across the groups over time thus cannot be drawn. The analysis is, however, useful for describing the broader mental health patterns observed in different samples drawn at different times from the population of interest and allows for a degree of macro-level inference (Firebaugh 1997). Sample sizes across the groups were also unequal and may have impacted the patterns observed, although adjustments for discrepancies in homogeneity of variance were statistically accounted for. The voluntary nature of participation in our study and limitations in the sampling strategies also makes it difficult to evaluate the representativeness of the samples. Further work with larger and more targeted samples that follows more traditional longitudinal models may broaden the understanding of mental health indicator patterns in the target population and trajectories over time. Research exploring the roles played by various demographic, contextual, and environmental factors in determining mental health indicator trajectories over time is also needed.

Overall, our findings suggest that there may be nuanced mental health patterns observed in physiotherapists over time during the COVID-19 pandemic. Gaining a better understanding of these may assist in the development of both targeted mental health interventions that account for both level of exposure to COVID-19 patients and the trajectory of the pandemic and training programmes that better equip physiotherapists to manage the demands of frontline work, especially during times of disruption. Our study presents a series of preliminary findings that can inform mechanisms to address the mental health profiles of physiotherapists over time, with a view to enhancing long-term retention and sustainability in the field.
